# Reality Monitoring and Feedback Control of Speech Production Are Related Through Self-Agency

**DOI:** 10.3389/fnhum.2018.00082

**Published:** 2018-03-06

**Authors:** Karuna Subramaniam, Hardik Kothare, Danielle Mizuiri, Srikantan S. Nagarajan, John F. Houde

**Affiliations:** ^1^Department of Psychiatry, University of California, San Francisco, San Francisco, CA, United States; ^2^Department of Radiology and Biomedical Imaging, University of California, San Francisco, San Francisco, CA, United States

**Keywords:** self-agency, reality monitoring, speech feedback monitoring, pitch perturbation, predicting self-generated action outcomes

## Abstract

Self-agency is the experience of being the agent of one’s own thoughts and motor actions. The intact experience of self-agency is necessary for successful interactions with the outside world (i.e., reality monitoring) and for responding to sensory feedback of our motor actions (e.g., speech feedback control). Reality monitoring is the ability to distinguish internally self-generated information from outside reality (externally-derived information). In the present study, we examined the relationship of self-agency between lower-level speech feedback monitoring (i.e., monitoring what we hear ourselves say) and a higher-level cognitive reality monitoring task. In particular, we examined whether speech feedback monitoring and reality monitoring were driven by the capacity to experience self-agency—the ability to make* reliable* predictions about the outcomes of self-generated actions. During the reality monitoring task, subjects made judgments as to whether information was previously self-generated (self-agency judgments) or externally derived (external-agency judgments). During speech feedback monitoring, we assessed self-agency by altering environmental auditory feedback so that subjects listened to a perturbed version of their own speech. When subjects heard minimal perturbations in their auditory feedback while speaking, they made corrective responses, indicating that they judged the perturbations as errors in their speech output. We found that self-agency judgments in the reality-monitoring task were higher in people who had smaller corrective responses (*p* = 0.05) and smaller inter-trial variability (*p* = 0.03) during minimal pitch perturbations of their auditory feedback. These results provide support for a unitary process for the experience of self-agency governing low-level speech control and higher level reality monitoring.

## Introduction

Self-agency is the experience of being the agent of one’s own thoughts and motor actions (Haggard, 2017; Korzyukov et al., 2017). The intact experience of self-agency is necessary for successful interactions with the outside world through reality monitoring. Reality monitoring is defined as the ability to distinguish the source of internally self-generated information from outside reality (externally-derived information; Johnson et al., 1993; Keefe et al., 1999; Vinogradov et al., 2008; Subramaniam et al., 2012). Reality monitoring is inextricably tied to successfully recognizing one’s own self-generated actions (self-agency; Bentall et al., 1991; Johnson et al., 1993; Morrison and Haddock, 1997; Keefe et al., 1999; Vinogradov et al., 2008; Subramaniam et al., 2012). In this study, we relate the experience of self-agency in a lower-level speech motor feedback experiment with a higher-level cognitive reality monitoring task. In particular, we examined whether speech feedback monitoring and reality monitoring were driven by the capacity to experience self-agency—the ability to make* reliable* predictions about the outcomes of self-generated actions.

## Self-Agency During Reality Monitoring

The experience of self-agency, a necessary component of reality monitoring, enables accurate judgments that “I generated my own actions” (Haggard, 2017; Korzyukov et al., 2017). This experience of self-agency results from the ability to reliably predict the outcomes of one’s own self-generated actions via successful encoding and memory retrieval of these self-generated actions. Predictions during reality monitoring require that subjects make conscious retrospective judgments regarding the source of information (i.e., subjects need to identify whether information was previously self-generated during judgments of self-agency or identify whether information was externally-derived during judgments of external-agency). Thus, the resulting experience of self-agency is thought to depend on people making reliable predictions regarding the outcomes of their self-generated actions based on recalling their past experiences so that they can use this prior information to update their current state during identification of this self-generated information in order to guide future predictions about their self-generated action outcomes.

## Self-Agency During Speech Feedback Monitoring

The experience of self-agency can also be observed during speech production experiments in which speakers monitor their auditory feedback while speaking (i.e., monitor what they hear themselves say). The predictions that participants make during speech feedback monitoring are largely automatic, unconscious and prospective, involving comparing incoming sensory feedback with predictions of that feedback (i.e., before and while listening to one’s own speech; Houde et al., 2002; Hickok et al., 2011; Houde and Nagarajan, 2011; Ford and Mathalon, 2012; Chang et al., 2013; Kort et al., 2014). Speakers experience self-agency only when auditory feedback minimally deviates from predictions of what they expect to hear (Korzyukov et al., 2017). When subjects hear such minimal perturbations in their auditory feedback while speaking, they typically make compensatory corrective responses that oppose the direction of perturbation, indicating that they judge the perturbations as errors in their speech output (Burnett et al., 1998; Houde et al., 2002; Liu and Larson, 2007; Houde and Nagarajan, 2011; Chang et al., 2013; Scheerer et al., 2013; Kort et al., 2014; Ranasinghe et al., 2017). In other words, speakers generally compensate for perturbations in auditory feedback to correct for what they perceive as errors in their speech output so that their actual speech output more closely matches their intended output (Hain et al., 2000). However, it remains to be understood whether these corrective responses are modulated by subjects’ reliance on internal predictions about the outcomes of their actions based on their prior experience of sensorimotor feedback (Kording and Wolpert, 2004). If increased reliance on internal predictions results in the experience of self-agency, we would expect that subjects who rely more on their internal predictions to guide their speech output, will consequently rely less on external auditory feedback, resulting in smaller compensatory corrective responses and an enhanced sense of self-agency that they followed their internal predictions to generate their own actions (i.e., their speech output).

The primary focus of this study is to examine whether there is a unitary experience of self-agency that is driven by the reliance in reliably predicting the outcomes of self-generated actions. This would then mean that self-agency during lower level speech feedback monitoring (indexed by smaller compensatory responses) would be correlated with self-agency during reality monitoring (indexed by accurate identification of self-generated information). We had two specific hypotheses: (1) The magnitude of corrective responses only during minimal pitch perturbations would negatively correlate with accurate self-agency judgments on the reality-monitoring task. In other words, we predicted that speakers who make smaller corrective responses to compensate for their errors during these minimal pitch-induced perturbations would manifest a greater sense of self-agency, reflecting their increased reliance on internal predictions to guide their speech output, rather than reliance on external altered feedback to influence their speech output. (2) Inter-trial variability in the magnitude of corrective responses only during minimal pitch-induced perturbations would negatively correlate with accurate self-agency judgments on the reality-monitoring task. In other words, we predicted that smaller inter-trial variability in the magnitude of a subject’s corrective responses during minimal pitch-induced perturbations would also indicate increased confidence in the subject’s sense of self-agency, and would correlate with enhanced accurate self-agency judgments on the reality-monitoring task.

## Materials and Methods

### Participants

In the present study, we recruited 19 healthy participants (9 female, 10 male, mean age = 27.26, mean education = 19.14). This study was approved by the Internal Review Board at the University of California San Francisco (UCSF). All participants gave written informed consent and then completed in the pitch perturbation task. Inclusion criteria for healthy participants were: no psychiatric/neurological disorders, no substance dependence or current substance abuse, good general physical health, age between 18 years and 60 years, right-handed and English as first language. One participant was unavailable to complete the reality-monitoring task.

### Pitch Feedback Perturbation Experiment

Subjects wore an over-the-ears microphone (AKG Pro Audio C520 Professional Head-Worn Condenser Microphone, AKG Acoustics, Vienna, Austria) and a pair of circumaural headphones. The microphone was connected to a pre-amplifier (specs) that was connected to a sound card (M-Audio Delta 44 4 × 4 analog I/O, M-Audio, Cumberland, RI, USA) in a computer (Dell OptiPlex 9020 Mini Tower, Dell Inc., Round Rock, TX, USA). The amplified audio signal was played back through the headphones.

The pitch perturbation experiment consisted of two runs (one 100 cents or 1/12th of an octave pitch perturbation run, and another 400 cents or 1/3rd of an octave pitch perturbation run) with 74 trials per run. Each trial began with a large green dot that appeared on the screen of the computer. Subjects were instructed to start vocalizing the vowel /a/ when they saw the green dot. They continued phonation for 2.4 s until the dot disappeared while listening to real-time auditory feedback via headphones. There was an inter-trial interval of 2.5 s.

In each trial, the phonation onset triggered a brief perturbation (of 100 cents for the minimal pitch-induced shift of 100 cents run or a larger shift of 400 cents for the 400 cents run) in the pitch of the subject’s feedback (Kort et al., 2014; Ranasinghe et al., 2017). Feedback perturbation was carried out by the computer using a vocoder process, and occurred with a randomly jittered delay (200–500 ms) from phonation onset that lasted for 400 ms. The direction of pitch-shift was either upward or downward and the distribution of these shifts was pseudo-random such that half the trials had a positive shift and the other half had a negative shift. This jittered perturbation and pseudo-random distribution minimized expectation/anticipatory bias, preventing participants from being able to anticipate either the onset or direction of the pitch shift.

### Pitch Perturbation Data Analyses

Participants’ speech data (microphone input) and the feedback audio data (headphones output) were recorded at a sampling rate of 11,025 Hz. Pitch data from all 19 subjects were included in the analysis. Using an autocorrelation-based pitch tracking method, time-courses of all trials were plotted (Parsons, 1987). Time intervals starting at 200 ms prior to perturbation onset and ending 1000 ms post-onset were extracted from these time-courses. Trials with incorrect pitch tracking or short utterances were weeded out as bad trials and excluded. The absolute pitch values in Hertz were converted to cents using the following formula:

Cents_(t)_ = 1200 log_2_(Hertz(_t_)/HertzRef) where Hertz(_t_) is the pitch value in Hertz at time = t and HertzRef is the reference pitch in Hertz which is the mean pitch in a window spanning 50 ms prior to perturbation onset to 50 ms after perturbation onset.

**Figure 1:**
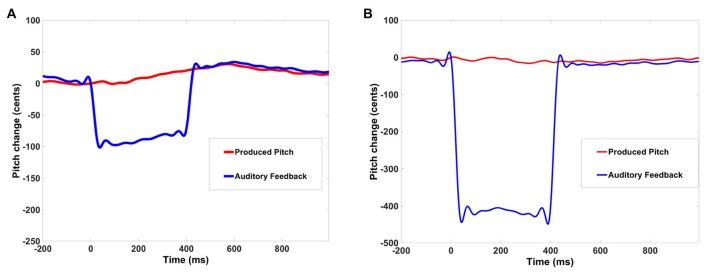
Example of one trial from one participant, in which the digital signal processing perturbed the participant’s vocal feedback by abruptly lowering the pitch for 400 ms by 100 cents **(A)** and by 400 cents **(B).** In response, the participant raised his/her pitch to partly compensate for the effects of the perturbation.

Participants responded to applied pitch perturbations by deviating from their baseline pitch track. For each participant, the mean of all pitch response tracks prior to perturbation was considered as the baseline pitch track. Responses in each trial were then computed as deviations from this baseline pitch track. To assay the magnitude of pitch deviations for each person, we computed the peak response in relation to the baseline pitch value (i.e., pitch prior to perturbation) in each trial and then computed the average peak response across trials, as we have performed in earlier studies (Chang et al., 2013; Ranasinghe et al., 2017). Figures 1A,B show examples of the peak response in one subject for 100 and 400 cents pitch perturbations. Following responses were defined as averaged pitch peak responses that were produced in the same direction and followed the auditory feedback of applied shift, yielding negative values. Compensatory corrective responses were defined as averaged pitch peak response produced opposing the direction of the auditory feedback of the applied shift, and yielded positive values. Specifically this means that if the pitch shift was downward, and the response was upward, this represents a positive compensatory response (as shown in Figure 1). Similarly, if the pitch shift was upward and the response was downward, this also represents a positive compensatory response. Conversely, if the pitch shift was downward and the response was also downward, this represents a negative compensatory response (i.e., following response). Therefore, the polarity of the corrective response was independent of the polarity of shift applied because it signified the direction of response and not the direction of shift. The peak values of the corrective responses were pooled together from both upward shift trials and downward shift trials. Consistent with our prior studies (Ranasinghe et al., 2017; Demopoulos et al., 2018), the method of analysis employed for this task looked at response deviations from the mean response track and thus the magnitudes of vocal responses were almost equidistant from the baseline and did not vary as a function of stimulus direction. To account for the trial-by-trial variability in peak response within participants, we computed the standard error of peak response for each participant. Standard error was computed as the standard deviation of peak response divided by the square root of the number of trials for each participant.

### Reality Monitoring Task

**Figure 2:**
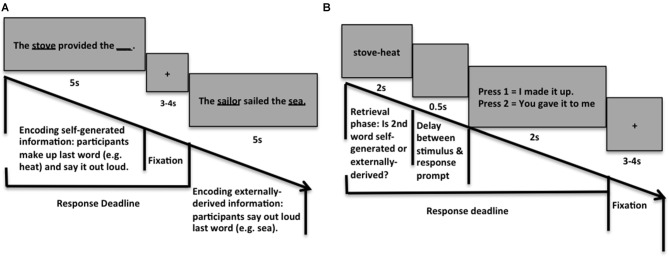
Reality Monitoring Task Design. **(A)** During encoding, participants were given sentences in which the final word was either left blank for participants to generate themselves (e.g., *The stove provided the __)* or was externally-derived as it was provided by the experimenter (e.g., *The sailor sailed the sea*). **(B)** During retrieval, participants were randomly presented with the noun pairs from the sentences (e.g., *stove-heat*), and had to identify with a button-press whether the second word was previously self-generated or externally-derived.

The reality-monitoring task consisted of an encoding phase and a memory retrieval phase (Figure 2). During encoding, participants were visually presented with semantically constrained sentences with the structure “noun-verb-noun”. On alternating half of the sentences, the final word was either left blank for participants to generate themselves (e.g., *The stove provided the __)* or was externally-derived as it was provided by the experimenter (e.g., *The sailor sailed the sea*). Altogether, participants were given 100 sentences for subjects to generate the final word (i.e., self-generated) sentences and 100 externally-derived sentences, presented in blocks of 20 trials per run. For each sentence, participants were told to pay attention to the underlined words and to vocalize the final word of each sentence (Figure 2A). Participants then completed the reality-monitoring retrieval task where they were randomly presented with the noun pairs from the sentences (e.g., *stove-heat*), and had to identify with a button-press whether the second word was previously self-generated or externally-derived (Figure 2B). The number of correctly identified self-generated and externally-derived trials was computed for each participant during the retrieval phase. Accurate self-agency judgments were computed for each subject as the percentage of correctly identified self-generated items out of the total number of self-generated trials during the retrieval phase of the task.

### Statistical Analyses

Repeated-measures ANOVAs were implemented to examine differences in peak deviation and inter-trial peak variability between 100 and 400 cents pitch perturbations, as well as between correctly identified self-generated and externally-derived information. Pearson’s two-tailed correlation tests were used to measure the strength of the linear relationship between peak deviation (for 100 cents and 400 cents pitch shifts) with reality-monitoring performance (self-generated, externally-derived accuracy). Effect sizes (Cohen’s d) were used to quantify the power of the linear relationships. Outliers were defined as values above/below 3 standard deviations from the mean. We did not find any outliers in any of the correlations. Mean RT was computed for correctly identified self-generated and externally-derived information.

## Results

**Figure 3:**
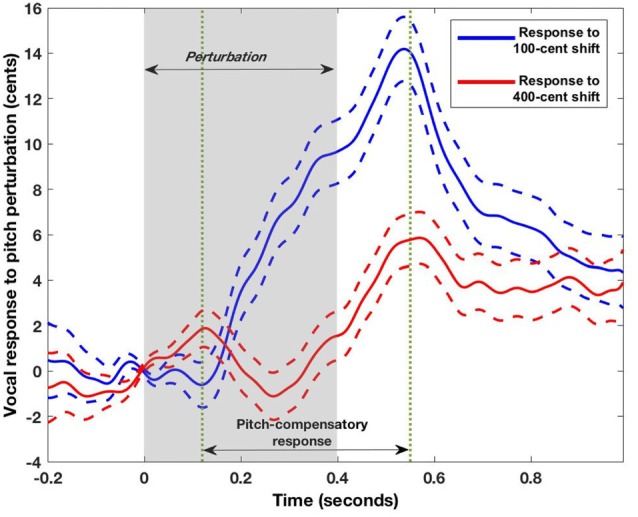
Mean pitch perturbation response tracks (±100 and ± 400) averaged across all participants (*N* = 19). The positive values of both contours indicate that the average response to both upward and downward shifts was compensatory. Participants began responding to the applied perturbation at an average of 120 ms after perturbation onset and peak response was attained at an average of 550 ms after perturbation. Dashed lines represent the standard error of the corrective responses across all trials and participants.

The behavioral responses to brief pitch perturbations in auditory feedback were mostly compensatory (i.e., opposing the direction of the applied shift) on both 100 cents (13 out of 19 participants made compensatory responses on average) and 400 cents (14 out of 19 participants made compensatory responses) experiments. In the example shown in Figure 1A from one participant, the digital signal processing perturbed the participant’s vocal feedback by abruptly lowering the pitch by 100 cents for 400 ms. In response, the participant raised his/her pitch to partly compensate for the effects of the perturbation. Participants began responding to the applied perturbation at an average of 120 ms after perturbation onset and peak response was attained at an average of 550 ms after perturbation (Figure 3). Consistent with previous studies, participants showed significantly smaller peak responses to the 400 cents pitch shift when compared to the 100 cents pitch shift (*F*_(1,18)_ = 8.92, *p* = 0.008; Figure 3; Behroozmand and Larson, 2011; Scheerer et al., 2013; Korzyukov et al., 2017). The behavioral response to pitch shift was variable from trial to trial in each participant. We found that participants also showed reduced inter-trial variability in their peak responses for the 400 cents pitch shift when compared to the 100 cents pitch shift (*F*_(1,18)_ = 28.01, *p* < 0.0001). We did not find any difference between 100 vs. 400 cents pitch perturbations in the time to reach peak magnitude from perturbation onset (*F*_(1,18)_ = 0.77, *p* = 0.39; see Table 1).

**Table 1:** Pitch production during perturbations.

	100 cents	400 cents	100 vs. 400 cents (*p* value)
Peak deviation (cents)	0.111 ± 0.16	0.011 ± 0.28	*p* = 0.008
Time to peak (seconds)	0.520 ± 0.15	0.559 ± 0.114	*p* = 0.39
Variability in peak deviation (cents)	0.062 ± 2.05	0.011 ± 0.004	*p* < 0.0001

**Figure 4:**
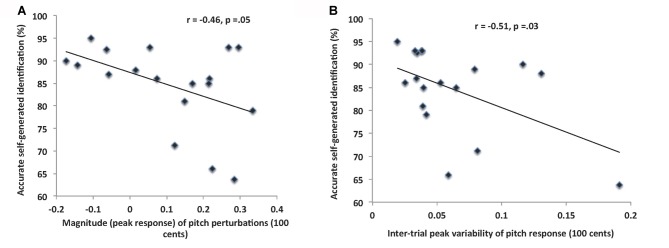
**(A)** The scatterplot illustrates the significant negative correlation between peak response to 100 cents pitch perturbations and judgments of self-agency on the reality monitoring task. **(B)** The scatterplot illustrates the significant negative correlation between the inter-trial variability in peak response to 100 cents pitch perturbations and judgments of self-agency on the reality-monitoring task.

In the reality monitoring task, participants were faster (*F*_(1,17)_ = 40.01, *p* < 0.0001) although not more accurate (*F*_(1,17)_ = 1.14, *p* = 0.30) at identifying self-generated information when compared to externally-derived information (see Table 2). Consistent with our hypothesis, for the 100 cents perturbations, we found a significant negative correlation between peak response to the pitch perturbations and correctly identified self-generated information on the reality-monitoring task (*r*_(16)_ = −0.46, *p* = 0.05; Figure 4A). In other words, speakers who made smaller corrective responses to compensate for their errors during 100 cents pitch-induced perturbations manifested a greater sense of self-agency during reality monitoring, reflecting their increased reliance on internal predictions to guide their speech output, rather than reliance on external altered feedback to influence their speech output.

**Table 2:** Reality-monitoring accuracy and reaction times (RT).

	Self-generated identification	Externally-derived identification	Self vs. External identification (*p* value)
Accuracy (%)	84.64 ± 9.25	79.71 ± 18.47	*p* = 0.30
RT (seconds)	1.22 ± 0.12	1.37 ± 0.16	*p* < 0.0001

Indeed, some subjects had compensatory responses that were so reduced that they were negative, which we refer to as following responses. Such following responses are thought to reflect speakers’ interpretation of the pitch shift as an external pitch target, rather than reflecting errors in their own speech output (Burnett et al., 1998; Hain et al., 2000). This is consistent with the idea that speakers who generate following responses rely more on their internal predictions to guide their speech output towards an external target (Burnett et al., 1998; Hain et al., 2000).

We did not observe any significant associations between 100 cents pitch perturbation responses and accuracy on identification of externally-derived information on the reality monitoring task. We also did not observe and significant associations between 400 cents pitch perturbations responses with either self-generated or externally-derived identification on the reality monitoring task (all *p*’s > 0.50). These results are consistent with our hypothesis, in which we expected to only find correlations between self-agency judgments during reality monitoring with the peak magnitude of deviation responses during minimal pitch-induced perturbations experienced with the 100 cents pitch shift, as only these minimal deviations in perturbations are thought to maintain the sense of self-agency.

We observed an even stronger negative correlation between the inter-trial variability in peak response to the 100 cents pitch perturbation with self-agency (i.e., indexed by accurate self-generated identification) during the reality-monitoring task (*r*_(16)_ = −0.51, *p* = 0.03; Figure 4B). This finding shows that subjects with smaller inter-trial variability during the 100 cents pitch perturbations manifested increased confidence in their self-agency judgments. As expected, we found no association in inter-trial variability in peak response during 100 cents with accurate identification of externally-derived information on the reality monitoring task. We also found no association in inter-trial peak response variability during the 400 cents shift with accurate identification of self-generated or externally-derived information on the reality monitoring task (all *p*’s > 0.40). Also, the length of time to reach peak magnitude from perturbation onset was not associated with either self or external agency judgments during reality monitoring, suggesting that only the size and inter-trial variability of perturbation responses provided reliable markers of self-agency judgments, and only during the 100 cents pitch perturbation. Finally, Cohen’s *d* analyses indicated that the correlation coefficients between self-generated accuracy during reality monitoring with peak perturbation size (Cohen’s *d* = 0.59) and variability (Cohen’s *d* = 0.48) during the 100 cents shift yielded large effect sizes. Together, these findings across converging different types of analyses provide support for our hypothesis that there is a unitary sense of self-agency that results from the ability to make* reliable* predictions about the outcomes of self-generated actions.

## Discussion

We found that when participants’ pitch was perturbed by 100 cents: (1) their peak perturbation responses were larger compared to when their pitch was perturbed by 400 cents; (2) their peak perturbation responses negatively correlated with accurate self-agency judgments (i.e., accurate self-generated identification) on the reality-monitoring task; and (3) the inter trial variability in their peak perturbation responses negatively correlated with their self-agency judgments on the reality-monitoring task.

Our findings are consistent with prior work indicating participants produce larger perturbation responses to 100 cents pitch perturbations than 400 cents pitch perturbations (Behroozmand and Larson, 2011; Scheerer et al., 2013; Korzyukov et al., 2017). This is because large pitch perturbations of 400 cents are thought to be registered as non self-generated (i.e., externally-derived) because of the unusually larger mismatch between internal predictions and auditory feedback, warranting minimal corrective responses (Liu et al., 2011; Korzyukov et al., 2017). By contrast, smaller pitch perturbations of 100 cents yield smaller mismatches between internal predictions and auditory feedback. These smaller mismatches are within the expected range of variation during normal speech production, and are interpreted as self-generated, and are thus more likely to generate corrective responses.

The principal hypothesis of this study was that there would be a significant association between peak perturbation responses to 100 cents pitch shifts and accurate self-agency judgments on the reality monitoring task. Consistent with this hypothesis, we found that participants who had smaller peak corrective responses only during 100 cents pitch shifts also manifested enhanced self-agency judgments (indexed by more accurate identification of self-generated information during reality-monitoring). This likely reflects their increased reliance on internal predictions to guide their speech output, rather than reliance on external altered feedback to influence their speech output. It must be noted that only the perturbations experienced during the 100 cents pitch perturbations (not the 400 cents perturbations) are interpreted as self-generated as only these minimal pitch perturbations are thought to maintain the sense of self-agency. As mentioned previously, larger pitch perturbations greater than 250–300 cents, are interpreted as non self-generated outcomes that are due to external changes in the environment rather than being reflective of judgments in self-agency (Behroozmand and Larson, 2011; Korzyukov et al., 2017). Thus, as expected, we did not find any significant correlations between peak perturbation responses during 400 cents pitch shifts with self-agency judgments during reality monitoring.

We also found that participants who had lower inter-trial peak variability also manifested more confidence and reliance in their judgments of self-agency during reality monitoring. Together, these findings indicate that self-agency judgments on the reality-monitoring task were highest in people who had the smallest peak perturbation response and the smallest inter-trial variability during 100 cents pitch shifts. These findings, therefore, indicate that the magnitude and inter-trial variability of responses to 100 cents pitch shifts provide quick and robust markers of the experience of self-agency, indicating which subjects followed their internal predictions to guide their own speech output.

In summary, our results are consistent with previous research (Hain et al., 2000; Behroozmand and Larson, 2011; Korzyukov et al., 2017) in that we expected subjects to generate corrective responses to compensate for the error in their speech output between the perceived and expected auditory feedback of their speech output particularly during minimal pitch perturbations. However, our findings take this prior research (Hain et al., 2000) one step further in that we demonstrated here that it was the gain in this prediction error modulated by subjects’ *confidence* in their own internal predictions about their speech outcome that determined whether they made larger or smaller compensatory corrective responses. In other words, the magnitude of compensatory responses was determined by subjects’ confidence levels, that modulates the feedback prediction error, in their speech outcome based on their prior experience of sensorimotor feedback (Kording and Wolpert, 2004). The more confident they were in their internal predictions about their speech outcomes, the less they relied on external auditory feedback, resulting in smaller corrective responses which correlated with improved conscious judgments of self-agency during reality monitoring.

It may be argued that one putative mechanism by which self-agency judgments are enhanced is via increased working memory, which mediates decreased vocal compensations and enhanced encoding and retrieval of self-generated and externally-derived information. However, if reduced corrective responses were mediated by enhanced working memory capacities, we would expect to find a significant correlation between the magnitude of corrective responses with external-agency judgments, which we did not find. Participants actually took longer to retrieve externally-derived information when compared to self-generated information, indicating that making external-agency judgments was more effortful, and thus required more working memory demand than making self-agency judgments. Rather, our data indicate that participants who had smaller peak corrective responses during 100 cents pitch shifts manifested enhanced judgments that were specific to self-agency (not external-agency), reflecting their increased reliance on internal predictions to guide their own actions (their speech output).

Taken together, our findings across converging different types of analyses provide evidence for the existence of a unitary sense of self-agency, and this results from the ability to make* reliable* predictions about the outcomes of self-generated actions. This is why measures of self-agency during lower level speech feedback monitoring correlated and predicted self-agency judgments (but not external-agency judgments) during higher-level reality monitoring.

## Conclusion

In conclusion, the sense of agency is critical for human beings to interact with the outside world. This is a first-of-its-kind study in which we clearly demonstrate how internal predictions regarding the outcomes of self-generated actions can modulate the experience of self-agency. These findings show that self-agency during lower-level speech feedback monitoring (i.e., indexed by smaller corrective responses during minimal pitch-induced perturbations) directly correlates with self-agency judgments during higher-level reality monitoring (i.e., indexed by more accurate identification of self-generated information. Taken together, our converging findings provide support for a unitary sense of self-agency that results from the ability to reliably predict the outcomes of self-generated actions.

These results, therefore, have important implications for both the assessment and enhancement of people’s experience in self-agency not only in healthy participants but also for patients suffering from psychosis-spectrum disorders (such as schizophrenia, schizoaffective disorder and bipolar disorder) who manifest severe deficits in judgments of self-agency. Future studies are needed to investigate both behavioral and neural responses to auditory feedback perturbations during speaking in healthy people as well as in patients with psychosis. This will help further validate the speech perturbation and reality monitoring paradigms as accurate assessments of the experience of self-agency.

## Author Contributions

KS recruited all the subjects and designed the reality monitoring experiment; acquired and analyzed the reality monitoring data; and interpreted the data and wrote and edited the manuscript. HK helped to design the speech monitoring experiment, acquired and analyzed the speech monitoring data. DM helped with recruitment of subjects and helped with designing and acquiring the reality monitoring data. SSN edited the manuscript, and provided advice on the acquisition, analyses and interpretation of the data; developed the speech monitoring experiment, and provided advice on the acquisition, analyses and interpretation of the data. JFH edited the manuscript.

## Conflict of Interest Statement

The authors declare that the research was conducted in the absence of any commercial or financial relationships that could be construed as a potential conflict of interest.
